# Sample size calculation based on exact test for assessing differential expression analysis in RNA-seq data

**DOI:** 10.1186/1471-2105-14-357

**Published:** 2013-12-06

**Authors:** Chung-I Li, Pei-Fang Su, Yu Shyr

**Affiliations:** 1 Department of Applied Mathematics, National Chiayi University, Chiayi, Taiwan; 2 Department of Statistics, National Cheng Kung University, Tainan, Taiwan; 3 Center for Quantitative Sciences, Vanderbilt University, 571 Preston Building Nashville, TN, USA

## Abstract

**Background:**

Sample size calculation is an important issue in the experimental design of biomedical research. For RNA-seq experiments, the sample size calculation method based on the Poisson model has been proposed; however, when there are biological replicates, RNA-seq data could exhibit variation significantly greater than the mean (i.e. over-dispersion). The Poisson model cannot appropriately model the over-dispersion, and in such cases, the negative binomial model has been used as a natural extension of the Poisson model. Because the field currently lacks a sample size calculation method based on the negative binomial model for assessing differential expression analysis of RNA-seq data, we propose a method to calculate the sample size.

**Results:**

We propose a sample size calculation method based on the exact test for assessing differential expression analysis of RNA-seq data.

**Conclusions:**

The proposed sample size calculation method is straightforward and not computationally intensive. Simulation studies to evaluate the performance of the proposed sample size method are presented; the results indicate our method works well, with achievement of desired power.

## Background

Next generation sequencing (NGS) technology has revolutionized genetic analysis; RNA-seq is a powerful NGS method that enables researchers to discover, profile, and quantify RNA transcripts across the entire transcriptome. In addition, unlike the microarray chip, which offers only quantification of gene expression level, RNA-seq provides expression level data as well as differentially spliced variants, gene fusion, and mutation profile data. Such advantages have gradually elevated RNA-seq as the technology of choice among researchers. Nevertheless, the advantages of RNA-seq are not without computational cost; as compared to microarray analysis, RNA-seq data analysis is much more complicated and difficult. In the past several years, the published literature has addressed the application of RNA-seq to multiple research questions, including abundance estimation [1-3], detection of alternative splicing [4-6], detection of novel transcripts [6,7], and the biology associated with gene expression profile differences between samples [8-10]. With this rapid growth of RNA-seq applications, discussion of experimental design issues has lagged behind, though more recent literature has begun to address some of the relevant principles (e.g., randomization, replication, and blocking) to guide decisions in the RNA-seq framework [11,12].

One of the principal questions in designing an RNA-seq experiment is: What is the optimal number of biological replicates to achieve desired statistical power? (Note: In this article, the term “sample size” is used to refer to the number of biological replicates or number of subjects.) Because RNA-seq data are counts, the Poisson distribution has been widely used to model the number of reads obtained for each gene to identify differential gene expression [8,13]. Further, [12] used a Poisson distribution to model RNA-seq data and derive a sample size calculation formula based on the Wald test for single-gene differential expression analysis. It is worth noting that a critical assumption of the Poisson model is that the mean and variance are equal. This assumption may not hold, however, as read counts could exhibit variation significantly greater than the mean [14]. That is, the data are over-dispersed relative to the Poisson model. In such cases, one natural alternative to Poisson is the negative binomial model. Based on the negative binomial model, [14,15] proposed a quantile-adjusted conditional maximum likelihood procedure to create a pseudocount which lead to the development of an exact test for assessing the differential expression analysis of RNA-seq data. Furthermore, [16] provided a Bioconductor package, edgeR, based on the exact test.

Sample size determination based on the exact test has not yet been studied, however. Therefore, the first goal of this paper is to propose a sample size calculation method based on the exact test.

In reality, thousands of genes are examined in an RNA-seq experiment; differential expression among those genes is tested simultaneously, requiring the correction of error rates for multiple comparisons. For the high-dimensional multiple testing problem, several such corrected measures have been proposed, such as family-wise error rate (FWER) and false discovery rate (FDR). In high-dimensional multiple testing circumstances, controlling FDR is preferable [17] because the Bonferroni correction for FWER is often too conservative [18]. Many methods have been proposed to control FDR in the analysis of high-dimensional data [17,19,20]. Those concepts have been extended to calculate sample size for microarray studies [21-25]. To our knowledge, however, the literature does not address determination of sample size while controlling FDR in RNA-seq data. Therefore, the second purpose of this paper is to propose a procedure to calculate sample size while controlling FDR for differential expression analysis of RNA-seq data.

In sum, in this article, we address the following two questions: (i) For a single-gene comparison, what is the minimum number of biological replicates needed to achieve a specified power for identifying differential gene expression between two groups? (ii) For multiple gene comparisons, what is the suitable sample size while controlling FDR? The article is organized as follows. In the Method section, a sample size calculation method is proposed for a single-gene comparison. We then extend the method to address the multiple comparison test issue. Performance comparisons via numerical studies are described in the Results section. Two real RNA-seq data sets are used to illustrate sample size calculation. Finally, discussion follows in the Conclusions section.

## Method

### Exact test

In an RNA-seq experiment, the total number of reads, also referred to as library size, mapped to the genome are different among the samples. In such cases, the counts in each group are not identically distributed, and it is difficult to develop an exact test for assessing the differential expression analysis of RNA-seq data. To handle this issue, [14,15] proposed a quantile-adjusted conditional maximum likelihood procedure to create pseudocounts which are approximately identically distributed and which lead to the development of an exact test. In the following, the proposed sample size calculation method is based the exact test for a single-gene comparison. Let *Y*_*ij*_ be the random variable corresponding to the pseudocount, with *y*_*ij*_ being the observed value of *Y*_*ij*_, of the *j*th (*j* = 1,2,…,*n*_*i*_) sample of the *i*th (*i* = 0,1) group where *n*_0_ and *n*_1_ are the numbers of samples from the control and treatment group, respectively. Assume pseudocount *Y*_*ij*_ can be modeled as a negative binomial (NB) distribution, NB(*d*_*ij*_*γ*_*i*_,*ϕ*). Here, *γ*_*i*_ represents the normalized gene expression level of group *i*, *d*_*ij*_ represents a normalization factor for the total number of reads mapped in the *j*th sample of the *i*th group, and *ϕ* is the dispersion. We use the NB parameterization where the mean is *μ*_*ij*_ = *d*_*ij*_*γ*_*i*_ and variance is $\mu_{\text{ij}}(1+\mu_{\text{ij}}^{2}\phi )$. Because the question of interest is to identify the differential gene expression between two groups, the corresponding testing hypothesis is

(1) $$H_{0}:\gamma_{1}=\gamma_{0}\;\;\mathrm{vs.}\;H_{1}:\gamma_{1}\neq \gamma_{0}.$$

Because the pseudocounts in each group have an approximately identical negative binomial distribution [14,15], the sum of pseudocounts of each group, $Y_{i}=\overset{n_{i}}{\underset{j=1}{\sum }}Y_{\text{ij}}$, has a negative binomial distribution NB($n_{i}d_{i}^{*}\gamma_{i},\phi /n_{i}$) where $d_{i}^{*}$ is the geometric mean of normalization factors in group *i*. Under the null hypothesis (1), the sum of the total pseudocount, *Y*_1_+*Y*_0_, follows a negative binomial distribution. In analogy with Fisher’s exact test, [14,15] proposed an exact test for replacing the hypergeometric probabilities with negative binomial probabilities. Because [16] developed a Bioconductor software package edgeR which is an implementation of methodology developed by [14,15], the *p*-value can be easily calculated for conducting the exact test.

In the following simulation and application sections, we used edgeR version 3.0.6 for estimating model parameters and performing the exact test.

### Sample size calculation for controlling type I error rate

In this section, we focus on sample size calculation based on the exact test for a single-gene comparison as described in the test statistics section. For simplicity, we assume the RNA-seq experiment uses a balanced design (i.e., *n*_0_ = *n*_1_ = *n*), which is a special but common case. The following method could be easily extended to the unbalanced case (i.e. let *n*_0_ = *n* and *n*_1_ = *kn* where *k* is a predetermined ratio of the sample size of the control group to the treatment group). In order to perform sample size calculations, it is necessary to construct a power function for the testing described above. The power of a test is the probability that the null hypothesis is rejected when the alternative hypothesis is true. Since the distribution of the exact test statistic under the alternative hypothesis is unknown, however, it is difficult to derive a closed-form expression of the power function. Instead of deriving the distribution of test statistic under the alternative hypothesis, [26] proposed a method to calculate the power for the exact test based on a given *p*-value. Here, we borrow their concept to calculate power. For a given *p*-value, *p*(*y*_1_,*y*_0_) where *y*_0_ and *y*_1_ are the observed pseudo-sums, described in the previous section, the power can be expressed as

$$\begin{array}{l} \xi (n,\rho ,\mu_{0},\phi ,w,\alpha )=\;\\ \;\overset{\infty }{\underset{y_{0}=0}{\sum }}\overset{\infty }{\underset{y_{1}=0}{\sum }}f\left(\mathrm{nw\rho}\mu_{0},\frac{\phi }{n}\right)f\left(n\mu_{0},\frac{\phi }{n}\right)I(\;p(\;y_{1},y_{0})<\alpha ),\; \end{array}$$

where $w=d_{1}^{*}/d_{0}^{*}$ is the ratio of the geometric means of normalization factors between two groups, *ρ* = *γ*_1_ / *γ*_0_ is the fold change, $\mu_{0}=d_{0}^{*}\gamma_{0}$ is the average number of reads in the control group, *f*(*μ*,*ϕ*) is the probability mass function of the negative binomial distribution with mean *μ* as well as dispersion *ϕ*, *α* is the the level of significance, and *I*(.) denotes the indicator function. For a given desired power 1-*β*, the power of the test can be represented as the function of sample size in the form

(2) $$\begin{array}{l} 1-\beta =\xi (n,\rho ,\mu_{0},\phi ,w,\alpha ).\; \end{array}$$

Thus, the required sample size *n* to attain the given power 1-*β* at level of significance *α* can then be calculated by solving (2) through a numerical approach, such as a gradient-search or bisection procedure.

### Sample size calculation for controlling false discovery rate

In reality, thousands of genes are examined in an RNA-seq experiment, and those genes are tested simultaneously for significance of differential expression. In such cases, the sample size calculation for a single-gene comparison discussed above cannot be applied directly. Jung, 2005 [23] incorporated FDR controlling based on a two-sample t-test under the Gaussian distribution assumption. In this section, we borrowed their concept to incorporate FDR controlling based on the test statistics described in the test statistics section.

For the multiple testing problem, [19] suggested the use of false discovery rate (FDR) which is defined as the expected proportion of false discoveries among rejected null hypotheses. Storey, 2002 [17] further proposed an improvement to FDR to achieve higher power, in the form

$$\text{FDR}=E\left(\frac{R_{0}}{R}\;|\;R>0\right),$$

where *R*_0_ is the number of false discoveries and *R* is the number of results declared significant (i.e., rejections of the null hypothesis).

To calculate the sample size for microarray data analysis, [23] proposed an FDR-controlled method which is based on the expression of FDR under independence (or weak dependence) among test statistics, as

$$\text{FDR}=\frac{m_{0}\alpha }{m_{0}\alpha +E(R_{1})},$$

[17,27], where *m*_0_ is the number of true null hypotheses and *E*(*R*_1_) is the expected number of true rejections. By borrowing their concepts, the expected number of true rejections for RNA-seq data can be calculated as

$$E(R_{1})=\underset{g\in M_{1}}{\sum }\xi (n,\rho_{g},\mu_{0g},\phi_{g},w,\alpha ),$$

where *ρ*_*g*_ is the fold change, *ϕ*_*g*_ is the dispersion, and *μ*_0*g*_ is the average read count in the control group for gene *g*∈*M*_1_ (the set of prognostic genes), respectively. Thus, to guarantee an expected number of true rejections, say *r*_1_, and control FDR at a specified level *f*, we have

(3) $$f=\frac{m_{0}\alpha }{m_{0}\alpha +r_{1}}$$

and

(4) $$r_{1}=\underset{g\in M_{1}}{\sum }\xi (n,\rho_{g},\mu_{0g},\phi_{g},w,\alpha ).$$

By solving equation (3) with respect to *α*, we have

$$\alpha^{*}=\frac{r_{1}\;f}{m_{0}(1-f)},$$

where *α*^∗^ is the marginal type I error level for the expected number of true rejections *r*_1_ at a given FDR *f*. Replacing *α* with *α*^∗^ in (4), we have the function with respect to *n* as

$$g_{1}(n)=\underset{g\in M_{1}}{\sum }\xi (n,\rho_{g},\mu_{0g},\phi_{g},w,\alpha^{*})-r_{1}.$$

Then, by solving *g*_1_(*n*) = 0 via a numerical approach, the required sample size for controlling FDR at level *f* can be obtained.

To calculate the sample size, we have to estimate all of the fold changes *ρ*_*g*_, dispersions *ϕ*_*g*_, and average read counts *μ*_0*g*_ of gene *g* for the set of prognostic genes *g*∈*M*_1_ prior to the RNA-seq experiment. However, we may not have enough information to estimate all of those parameters in practice. To address this issue, we propose the following method to obtain a conservative estimate of the required sample size. Because the power increases as | log2(*ρ*_*g*_)| or *μ*_0*g*_ increases and *ϕ*_*g*_ decreases, we suggest using a common $\rho^{*}=\arg\underset{g\in M_{1}}{\min}\{|\underset{2}{\log}(\rho_{g})|\}$ minimum fold change, $\mu_{0}^{*}=\underset{g\in M_{1}}{\min}\{\mu_{0g}\}$ minimum average read count, and $\phi^{*}=\underset{g\in M_{1}}{\max}\{\phi_{g}\}$ maximum dispersion to estimate each *ρ*_*g*_, *μ*_0*g*_, and *ϕ*_*g*_, respectively. In such cases, it gives a more conservative estimate of the required sample size.

When we use *ρ*^∗^, $\mu_{0}^{*}$, and *ϕ*^∗^ to estimate each *ρ*_*g*_, *μ*_0*g*_, and *ϕ*_*g*_, *g*∈*M*_1_, in the multiple testing context, *α*^∗^ and *β*^∗^ can be calculated as *r*_1_*f*/(*m*_0_(1-*f*)) and 1-*r*_1_/*m*_1_, respectively, where *m*_1_ is the number of prognostic genes. In other words, the power function (2) can be applied in the case of multiple gene comparison, with the replacement of *α* and *β* with *α*^∗^ and *β*^∗^.

The procedures for sample size calculation detailed in this section can be summarized as follows: 

1. Specify the following parameters: *m* : total number genes for testing; *m*_1_ : number of prognostic genes; *r*_1_ : number of true rejections; *f* : FDR level; *w* : ratio of normalization factors between two groups; {*μ*_0*g*_,*g*∈*M*_1_} : average read counts for prognostic gene *g* in control group; {*ρ*_*g*_,*g*∈*M*_1_} : fold changes for prognostic genes *g* in control group; {*ϕ*_*g*_,*g*∈*M*_1_} : dispersion for prognostic genes *g* in control group;

2. Calculate sample size: 

(a) If all the parameters *μ*_0*g*_, *ρ*_*g*_, and *ϕ*_*g*_ for each prognostic gene *g* are known, use a numerical approach to solve the equation below with respect to *n*.

$$r_{1}=\underset{g\in M_{1}}{\sum }\xi (n,\rho_{g},\mu_{0g},\phi_{g},w,\alpha^{*}),$$

where *α*^∗^ = *r*_1_*f*/(*m*_0_(1-*f*)) and *m*_0_ = *m*-*m*_1_;

(a) Otherwise, 

•••specify a desired minimum fold change *ρ*^∗^, a minimum average read count $\mu_{0}^{*}$, and a maximum dispersion *ϕ*^∗^;

•••replace *ρ* = *ρ*^∗^, $\mu_{0}=\mu_{0}^{*}$, *ϕ* = *ϕ*^∗^, *α* = *r*_1_*f*/(*m*_0_(1-*f*)), and *β* = 1-*r*_1_/*m*_1_ in equation (2) and solve it with respect to *n*.

## Results

### Numerical studies

In this section, we conducted simulation studies to evaluate the accuracy of the proposed sample size formula. The parameter settings in simulation studies are based on empirical data sets.

We set the total number of genes for testing to be *m* = 10000 and the number of statistically significant prognostic genes *m*_1_ = 100. We wanted to detect the expected number of true rejections *r*_1_ = 80, which corresponds to a power of 80% (i.e. *β*^∗^ = 0.2). All parameters *μ*_0*g*_, *ρ*_*g*_, and *ϕ*_*g*_ (*g* = 1,…,10000) were assumed to be unknown. Thus, we used a minimum fold change *ρ*^∗^ and a minimum average read count $\mu_{0}^{*}$ and a maximum dispersion *ϕ*^∗^ to estimate each *ρ*_*g*_, *μ*_0*g*_, and *ϕ*_*g*_, *g* = 1…,10000. We varied $\mu_{0}^{*}=1$ or 5; log_2_-fold changes log2(*ρ*^∗^) = 0.5,1.0,1.5,2.0 or 2.5; and *ϕ*^∗^ = 0.1, or 0.5. With these settings, *α*^∗^ = 8.162×10^-5^,4.253×10^-4^, and 8.979×10^-4^, which correspond to controlling FDR at level 1%, 5%, and 10%, respectively.

Then, we substituted *α*^∗^ and *β*^∗^ into the formulas (2) and calculated sample size by solving this equation. In addition, for each design setting, we generated 5000 samples from independent negative binomial distributions based on the calculated sample size *n*; for the control group, the count of each gene is generated by R program from a negative binomial distribution with mean $\mu_{0}^{*}$ and dispersion *ϕ*^∗^; for the treatment group, the count of each gene is generated from a negative binomial distribution with mean $\rho^{*}\mu_{0}^{*}$ and dispersion *ϕ*^∗^. Then, edgeR is used to estimate model parameters and perform the exact test. The number of true rejections was counted using the q-value procedure proposed by [20]. The expected number of true rejections was estimated as the sample mean of the number of rejections of the 5000 simulation samples (${\hat{r}}_{1}$).

In Table 1, we showed the calculated sample size with corresponding ${\hat{r}}_{1}$ in parentheses under the case *w* = 1. For a fixed log_2_-fold change, dispersion, and FDR, sample size increases when *μ*_0_ decreases. This result is as expected; a small average read count provides less information, such that a larger sample size is required to detect the difference. For a fixed $\mu_{0}^{*}$, *ϕ*^∗^, and FDR, sample size increases when log2(*ρ*^∗^) decreases (i.e. the smaller log_2_-fold changes requires greater sample sizes with all else being equal). This result is as expected; a larger sample size is required for detecting a smaller difference. For a fixed $\mu_{0}^{*}$, log2(*ρ*^∗^), and FDR, sample size increases when *ϕ*^∗^ increases. This result, also, is as expected; the variation increases when dispersion increases, such that a larger sample size is required to detect the difference. Note that all ${\hat{r}}_{1}$ in Table 1 are close to the pre-specified number of true rejections (*r*_1_=80); thus, the proposed method estimated a sample size that achieves correct power at the specified FDR level.

**Table 1:** **Sample size calculation for simulation study (and**${\hat{r}}_{1}$**) with** ***r***_**1**_** = 80** at FDR = **1%**, **5%** and **10%when** ***w* = 1,** ***m* = 10000,** ***m***_**1**_** = 100**

			$\mu_{0}^{*}=1$			$\mu_{0}^{*}=5$	
			**FDR**		**FDR**		
**log**_**2**_**(*ρ***^**∗**^**)**	***ϕ***^**∗**^	**1%**	**5%**	**10%**	**1%**	**5%**	**10%**
0.5	0.1	365 (81)	305 (84)	278 (88)	104 (81)	87 (84)	79 (88)
	0.5	518 (81)	433 (84)	394 (88)	257 (81)	215 (84)	196 (89)
1.0	0.1	79 (81)	67 (84)	61 (87)	24 (82)	20 (84)	19 (91)
	0.5	119 (81)	99 (83)	91 (88)	63 (82)	53 (85)	48 (89)
1.5	0.1	31 (82)	26 (83)	24 (86)	10 (83)	9 (90)	8 (91)
	0.5	49 (81)	41 (83)	38 (88)	28 (83)	23 (84)	21 (86)
2.0	0.1	16 (85)	13 (84)	12 (86)	6 (90)	5 (92)	4 (86)
	0.5	26 (82)	22 (84)	20 (86)	16 (84)	13 (85)	12 (89)
2.5	0.1	8 (85)	7 (89)	6 (87)	3 (78)	3 (81)	3 (98)
	0.5	14 (83)	12 (87)	11 (84)	10 (82)	9 (90)	8 (91)

### Applications

#### Liver and kidney RNA-seq data set

To identify differentially expressed genes between human liver and kidney RNA samples, [8] explored an RNA-seq data set containing 5 human kidney samples and 5 human liver samples. In the following, we used this data set as pilot data for designing a new study with the same study objective. For the purpose of demonstration, we assumed that the human kidney is the control group. After filtering genes with no more than 5 total reads in liver samples or kidney samples, there were 17306 genes left. We assumed that the top 175 (≈ 1% of 17306) genes are prognostic. From the pilot data, the minimum average read counts among the prognostic genes in the control group were estimated as $\mu_{0}^{*}=5$, the maximum dispersion was estimated as *ϕ*^∗^ = 0.0029, and the ratio of the geometric mean of normalization factors between the two groups was estimated as *w* = 0.9 using edgeR. Suppose we want to identify 80% of the prognostic genes (i.e. *r*_1_ = 0.8×175 = 140), while controlling FDR at 1% (i.e. *f* = 0.01). Based on the pilot data, we set *m* = 17306, *m*_1_ = 175, *m*_0_ = 17131, *r*_1_ = 140, and *f* = 0.01. In this case, we have

$$\begin{array}{lll} \alpha^{*} & =\frac{r_{1}\;f}{m_{0}(1-f)}\; & \;\\ & =8.2549\times 10^{-5}\; & \; \end{array}$$

and

$$\begin{array}{lll} \beta^{*} & =1-\frac{r_{1}}{m_{1}}\; & \;\\ & =0.2.\; & \; \end{array}$$

After substituting those parameters into equation (2) and solving it with respect to *n*, the required sample size can be obtained. In the second column from the left of Table 2, we report the sample size while controlling FDR at 1% under various desired minimum fold changes *ρ*^∗^ = 0.10,0.25,0.50,0.75,1.25,1.50,2.00,2.50, and 3.0. From Table 2, we found that the original RNA-seq experiment described in [8] with sample size 5 in each group can identify 80% of the prognostic genes at FDR =1*%* if the desired minimum fold change *ρ*^∗^ is 3.0.

**Table 2:** **Sample size calculation for liver and kidney RNA-seq data set under various desired minimum fold changes (*ρ***^***∗***^**) for** ***r***_**1**_** = 140at** ***F*** ***D*** ***R* = 1%when** ***m* = 17360and** ***m***_**1**_** = 175**

		**NB**		**Poisson**					
***ρ***^**∗**^		***n***		***n***_***w***_	***n***_***s***_	***n***_***lw***_	***n***_***ls***_	***n***_***tp***_	***n***_***lr***_
0.10		7		7	7	11	5	5	7
0.25		11		11	11	13	9	9	10
0.50		30		29	30	31	28	27	29
0.75		139		134	136	137	133	132	135
1.25		178		175	173	174	174	177	181
1.50		50		49	48	49	48	50	50
2.00		15		15	15	15	14	16	15
2.50		8		8	8	8	7	8	8
3.00		5		5	5	6	5	6	5

Li, 2013 [28] proposed several sample size calculation methods for RNA-seq data under the Poisson model. To compare the difference in sample size calculation between the negative binomial method and Poisson method, in the last six right columns of Table 2 we report the sample size calculation based on Poisson model (i.e. the sample size based on the Wald test *n*_*w*_, score test *n*_*s*_, log transformation of Wald statistic *n*_*lw*_, log transformation of score statistic *n*_*ls*_, transformation of Poisson *n*_*tp*_, and likelihood ratio test *n*_*lr*_) with the same settings as the negative binomial method. As we can see, the sample size calculation based on the negative binomial and Poisson methods are similar. This result is as expected since the data set explored by [8] has technical and not biological replicates (i.e. the maximum dispersion estimated from the liver and kidney RNA-seq data set is close to zero). Thus, it is not surprising that the results of the negative binomial and Poisson methods are similar when the dispersion parameter is close to zero. Moreover, in Table 2, the estimated sample size is about the same size for very small fold changes (*ρ*^∗^ = 0.10) and very large fold changes (*ρ*^∗^ = 3.0). This result is expected since it tends to the same conclusion no matter what statistical model is used when the treatment effect is very large (i.e. the fold change is very large or small).

#### Transcript regulation data set

Blekhman, 2010 [29] used RNA-seq to study transcript regulation in humans, chimpanzees, and rhesus macaques using liver RNA samples from three males and three females from each species. For the purpose of demonstration, we assumed that the goal of the study is to identify differential gene expression between male and female in humans and that the female is considered the control group. There were 13267 genes in the data set after performing quality control analyses. Suppose that the top 133 (≈ 1% of 13267) genes are prognostic. After filtering genes with no more than 5 total reads in male samples or female samples, there were 7658 genes left. Those genes are considered pilot data, and we assessed the differential expression by using edgeR. From the pilot data, the minimum average read counts among the prognostic genes in the control group were estimated as $\mu_{0}^{*}=1.67$, *ϕ*^∗^ = 0.6513, and the ratio of the geometric mean of normalization factors between the two groups was estimated as *w* = 1.08. Suppose we want to identify 80% of the prognostic genes (i.e. *r*_1_ = 0.8×133 = 107), while controlling the FDR at 10%. Based on the pilot data, we set *m* = 13267, *m*_1_ = 133, *m*_0_ = 13134, *r*_1_ = 107 and *f* = 0.1. In this case, we have *α*^∗^ = 9.0512×10^-4^ and *β*^∗^ = 0.2. In the second column from the left of Table 3, we report the required sample sizes under various desired minimum fold changes while controlling the FDR at 10% under the negative binomial distribution. We also report the required sample size based on the Poisson model proposed by [28] under the same settings in the last six columns on the right of Table 3. As we can see, the required sample size based on the negative binomial method is greater than the Poisson method. In the transcript regulation data set, the maximum dispersion was estimated as *ϕ*^∗^ = 0.6513>0. This indicates that the read counts in this data set exhibit over-dispersion. In such a situation, it is inappropriate to model this data set based on the Poisson, and the sample size calculation based on the Poisson model will be underestimated due to underestimation of variance (i.e. the study based on the corresponding sample size will be underpowered).

**Table 3:** **Sample size calculation for transcript regulation data set under various desired minimum fold changes (*ρ***^***∗***^**) for** ***r***_**1**_** = 107at** ***F*** ***D*** ***R* = 10%when** ***m* = 13267and** ***m***_**1 = 133**_

		**NB**		**Poisson**					
***ρ***^**∗**^		***n***		***n***_***w***_	***n***_***s***_	***n***_***lw***_	***n***_***ls***_	***n***_***tp***_	***n***_***lr***_
0.10		19		15	14	21	10	10	14
0.25		35		23	23	26	19	19	21
0.50		109		62	60	62	58	56	59
0.75		558		284	281	282	280	273	281
1.25		821		316	363	366	360	371	381
1.50		240		100	102	103	99	105	105
2.00		79		30	31	32	29	32	32
2.50		44		16	16	18	15	17	16
3.00		30		10	11	12	9	11	10

## Discussion

In this research, we assume independent gene expression levels; however, this assumption may not hold in reality. For correlated RNA-seq gene expression data, evaluation of the accuracy of our method is an important future research question; however, generating a negative binomial distribution for correlated high-dimensional data will be a challenge. Moreover, most of the major R packages dedicated to RNA-seq differential analyses (edgeR, DESeq, etc.) are now starting to enable multi-group comparisons. However, the proposed method is developed for comparing two-group means. Thus, the sample size calculation for multi-group comparisons would be an interesting research topic for us in the future. In addition, it has already been noted that typical RNA-seq differential analyses have very low power; see for example the simulation studies in [30], where power for edgeR was always less than 60%, or [31], where power ranged from about 45% to 55% (both with 10 samples per condition). In our simulation and application sections, the minimum sample sizes required to achieve 80% power would be prohibitively large for RNA-seq experiments in practice, given their current cost. In such situations, the findings in [30,31] can provide useful information for specifying achievable power. It is well known that low study power will decrease the reproducibility of scientific research. We hope that this paper can benefit researchers by allowing them to understand their study power.

## Conclusions

In recent years, RNA-seq technology has emerged as an attractive alternative to microarray studies, due to its ability to produce digital signals (counts) rather than analog signals (intensities), and to produce more highly reproducible results with relatively little technical variation [32,33]. With a large sample size, RNA-seq can become costly; on the other hand, insufficient sample size may lead to unreliable answers to the research question of interest. To manage the trade-off between cost and accuracy, sample size determination is a critical issue for RNA-seq experimental design. For comparing the differential expression of a single gene, we have proposed a sample size calculation method based on an exact test proposed by [14,15]. To address multiple testing (i.e., multiple genes), we further extended our proposed method to incorporate FDR control. Our methods are not computationally intensive for pilot data or other relevant data with a specified desired minimum fold change, minimum average read count, and maximum dispersion. To facilitate implementation of the sample size calculation, R code is available from the corresponding author.

## Competing interests

The authors declare that they have no competing interests.

## Authors’ contributions

Authors CIL, and PFS were involved in the development of the models. CIL and PFS wrote the manuscript. SY generated the original idea and guided and supervised the research. All authors read and approved the final version of this manuscript.
